# Negative Effects of an Exotic Grass Invasion on Small-Mammal Communities

**DOI:** 10.1371/journal.pone.0108843

**Published:** 2014-09-30

**Authors:** Eric D. Freeman, Tiffanny R. Sharp, Randy T. Larsen, Robert N. Knight, Steven J. Slater, Brock R. McMillan

**Affiliations:** 1 Brigham Young University, Department of Plant and Wildlife Sciences, Provo, Utah, United States of America; 2 Monte L. Bean Life Science Museum, Provo, Utah, United States of America; 3 United States Army Dugway Proving Ground, Environmental Programs, Dugway, Utah, United States of America; 4 HawkWatch International, Conservation Director, Salt Lake City, Utah, United States of America; Beijing Forestry University, China

## Abstract

Exotic invasive species can directly and indirectly influence natural ecological communities. Cheatgrass (*Bromus tectorum*) is non-native to the western United States and has invaded large areas of the Great Basin. Changes to the structure and composition of plant communities invaded by cheatgrass likely have effects at higher trophic levels. As a keystone guild in North American deserts, granivorous small mammals drive and maintain plant diversity. Our objective was to assess potential effects of invasion by cheatgrass on small-mammal communities. We sampled small-mammal and plant communities at 70 sites (Great Basin, Utah). We assessed abundance and diversity of the small-mammal community, diversity of the plant community, and the percentage of cheatgrass cover and shrub species. Abundance and diversity of the small-mammal community decreased with increasing abundance of cheatgrass. Similarly, cover of cheatgrass remained a significant predictor of small-mammal abundance even after accounting for the loss of the shrub layer and plant diversity, suggesting that there are direct and indirect effects of cheatgrass. The change in the small-mammal communities associated with invasion of cheatgrass likely has effects through higher and lower trophic levels and has the potential to cause major changes in ecosystem structure and function.

## Introduction

Exotic invasive species can directly and indirectly influence natural ecological communities by modifying structure [1], decreasing diversity [2], and altering ecosystem function [3]. Specifically, exotic plant species alter the hydrology of ecosystems, increase soil erosion, decrease native plant diversity, and alter fire cycles [4], [5]. Examples in North America include salt cedar (*Tamarix ramosissima*), kudzu (*Pueraria lobata*), leafy spurge (*Euphorbia esula*), red brome (*Bromus rubens*), and cheatgrass (*Bromus tectorum*). Alternatively, some exotic species can be benign or have net positive effects on systems where they are introduced or become established [6]. As exotic species continue to successfully invade and transform ecosystems, understanding the effects of these invasions on all trophic levels will be important for mitigating negative impacts, maintaining ecosystem integrity, preserving biodiversity, and predicting the consequences of further invasions [7], [8].

Cheatgrass invades and impacts communities worldwide [9], but has had particular success in the Great Basin Desert(s) of the western United States. In this ecosystem, cheatgrass has altered the fire cycle, outcompeted native vegetation, and altered ecosystem dynamics [10], [11], [12]. Remotely sensed data indicate that at least 40,000 square kilometers of the Great Basin (nearly 10%) are dominated by monocultures of cheatgrass [13]. Additionally, cheatgrass is a major understory component across a larger area and 200,000 additional square kilometers are vulnerable to invasion [14]. This invasion is noteworthy and concerning when considering that as little as 200 years ago, cheatgrass was isolated to a single population on the East coast and that the earliest records of cheatgrass in the Great Basin were from around 1900 [12], [15].

The invasion of this robust annual and other *Bromus* spp. is associated with increased frequency of fire and decreased plant diversity across much of the western US [16]. As cheatgrass invades a desert system, it fills the inter-plant spaces that normally separate native plant species. Where fires were once confined to relatively small areas because of limited connectivity, cheatgrass allows fire to carry over larger areas, impacting the native plant community more widely and frequently. In addition to increasing pressures on native plants through fire, cheatgrass often out-competes native plants through higher rates of root growth, seedling germination, and adult survival [17], [18].

Dramatic changes to the composition and structure of plant communities subjected to invasion often have cascading effects at higher trophic levels [19], [20], [21]. For example, invasive plant species in western North American grasslands alter predator-prey interactions (and subsequent survival of native species at higher trophic levels) by changing the structure of the physical environment and the abundance and diversity of native plants [22]. Specifically, a bottom-up effect occurs as cheatgrass invasion changes the availability of native plant resources used for forage and cover by a variety of taxa (e.g., small mammals) [23], [24]. Desert small mammals are primarily granivorous and prefer native seed over that of cheatgrass, which is nutritionally inferior [25]. Additionally, different species of small mammals forage in different microhabitats [26], [27], the variety of which is reduced when cheatgrass invades, fills inter-plant spaces, alters natural communities, and increases the frequency of fire [24]. Changes to habitat and food resources that result from cheatgrass invasion likely affect small-mammal communities and subsequently, higher trophic levels (e.g., canids and raptors).

As a keystone guild in North American deserts, granivorous small mammals drive and maintain plant diversity [28], [29]. This top-down effect occurs as granivorous small mammals modify the availability of reproductive propagules via seed gathering, caching, and consumption behaviors, which vary among species [26]. Seed caching behaviors of small mammals make them an important vector of dispersal for plants [30]. Because different species of small mammal have different consumption and caching behaviors, a change in the rodent community likely affects seed survival, dispersal, and plant recruitment. The potential impact of cheatgrass invasion on small-mammal communities may also have indirect effects on native plant diversity.

Our objective was to assess the effects of invasion by cheatgrass on small-mammal communities. To make this assessment, we determined abundance and diversity of small mammals at sites with varying levels of invasion by cheatgrass across the Great Basin Desert in northwest Utah. We predicted that as the percentage of cheatgrass cover increased: 1) the overall abundance of small mammals would decrease, but the responses of individual functional groups and species would vary, and 2) the species diversity (or indices of richness and evenness) of the small-mammal community would decrease. Because small-mammal assemblages often have significant effects on other trophic levels (e.g., primary producers and predators) and ecosystem processes (e.g., seed dispersal/consumption and soil disturbance), understanding the impact of invasion by cheatgrass on small-mammal communities is pertinent to the conservation of ecosystem structure and function.

## Methods

### Study site location and selection

**Figure 1:**
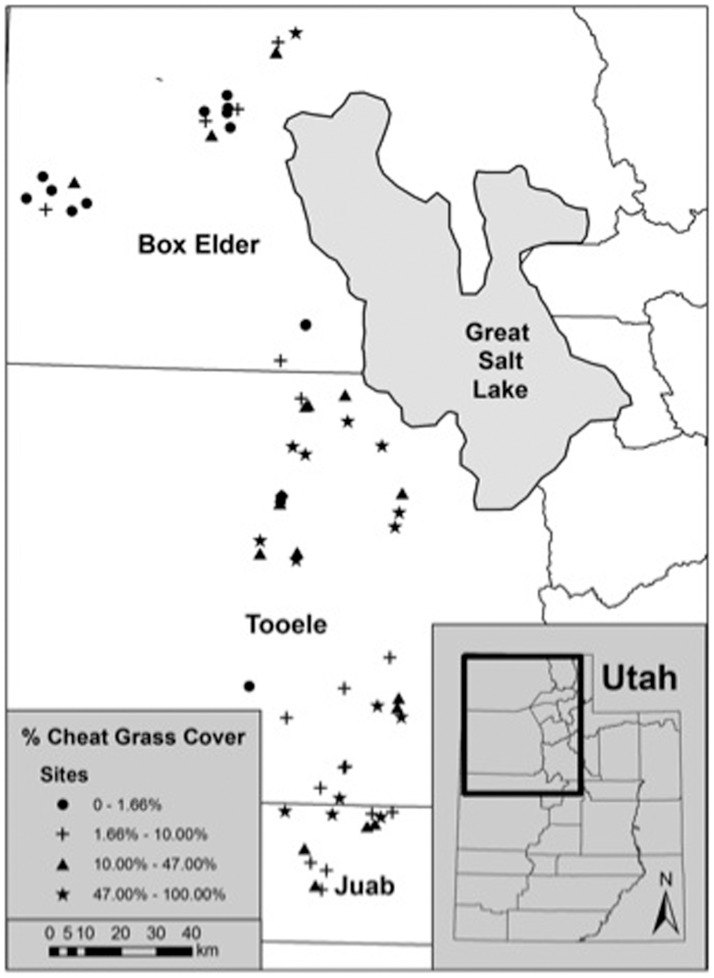
Study Area. Sites sampled for small mammals and the percentage of cheatgrass (*Bromus tectorum*) cover in the Great Basin Desert, Utah during the summers of 2011 and 2012. We divided sites into quartiles based on the percentage of cheatgrass cover.

During the summers of 2011 and 2012, we sampled small-mammal and plant communities at 70 sites across the Great Basin Desert in Box Elder, Tooele, and Juab Counties, Utah (Figure 1). These sites were located between 41°43′ N – 39°40′ N (North-South) and 113°57′ W – 112°39′ W (East-West). Plant communities were dominated by sagebrush (*Artemesia* spp.), saltbush (*Atriplex* spp.), greasewood (*Sarcobatus vermiculatus*), or cheatgrass, if lacking a shrub layer. We established a 90-m by 90-m trapping grid at each site, which were separated by at least 500 m.

We used remotely sensed MODIS and SWReGAP vegetation data and field observations made in 2011 of cheatgrass cover to select sites for sampling. We identified areas that differed in extent of cheatgrass invasion (low, medium or high) to ensure that we sampled sites across a continuum of cheatgrass cover. Within these areas, we established sites at computer-generated random points with an distribution across the levels of invasion. When generating random points, we only queried areas that were relatively close to roads (to ensure access) and requested a clumped distribution (separated by <50 km) for sampling of nearby sites within each week. This ensured that long-distance transportation between sites was limited so that traps were checked in a timely manner each morning. We selected and sampled 70 sites in a stratified random manner to avoid confounding level of invasion with time (small mammals may be less active at some times of the year).

### Plant survey

At each site sampled for small mammals, we established three 50-m transects that ran through the trapping grid, paralleling the 2^nd^, 4^th^, and 6^th^ lines of traps. We used a point-intercept method and sampled at 50-cm intervals along each transect. For each sample, we recorded if cheatgrass was present. The percentage of cheatgrass cover was estimated as the number of points with cheatgrass present divided by the total number of points at a site (300). Similarly, we recorded other plant species (or bare ground) present at each point and estimated the percentages of herbaceous plant and shrub cover in a similar manner. We used shrub cover as a proxy for fire (an indirect effect of cheatgrass) because burned sites in Great Basin shrub communities (which includes all of the sites we sampled) generally have reduced or no shrub cover, while shrubs are a major part of the plant community at unburned sites [12].

### Small mammal survey

We sampled small mammals at each site for three consecutive nights during the summers of 2011 and 2012. To sample small mammals, we established a 7×7 trapping grid at each site with 15 m spacing between trap stations and placed one trap at each station (49 traps; 90 m×90 m trapping grid). We used 7.6 cm×7.6 cm×30.5 cm collapsible Sherman live traps baited with commercially available birdseed. We checked traps each morning and closed them until evening if daytime temperatures were expected to exceed 22°C. We added cotton batting to the traps if nighttime lows were projected below 4.5°C. We took these precautions to decrease the likelihood of temperature-induced stress and to reduce incidental mortality. We identified small mammals captured to species and collected basic live-trap data (e.g., sex, age, mass, anatomical measurements, and reproductive condition). We temporarily marked all animals by shaving a small patch of fur and released them at the capture site. All capture and handling methods were approved by the Institutional Animal Care and Use Committee (IACUC) of Brigham Young University (Protocol Numbers 110306 and 120601). Additionally, we acquired a Certificate of Registration (# 1COLL8652) from the Utah Division of Wildlife Resources (permission to trap) and obtained permission to access lands whenever needed. Our study did not involve endangered or threatened species.

### Data analysis

We used linear regression analyses to assess relationships between factors potentially impacted by cheatgrass invasion (diversity of the plant and small-mammal community, the abundance of small mammals, and the percentage of shrub cover) and the percentage of cheatgrass cover. Given the structure and type (count) of our small mammal data, we used negative binomial distributions for the error structure in these analyses [31]. In other cases (e.g., for diversity indices and the percentage of shrub cover), we examined distributions and utilized a normal error structure (glm function in R). We calculated the minimum number of small mammals known to be alive (MNA; the total number of unique individuals captured) for each species at each site. We then examined the overall abundances of small mammals relative to the percentage of cheatgrass cover at each site using simple linear regression. We used the glm.nb function within the MASS package of program R [32] for these analyses [33]. Each regression model included sampling year to account for annual differences in small-mammal communities.

To better understand any ecological relationship illustrated in the overall regression, we also evaluated trends in abundance when the data were partitioned by family. We captured individuals from the Families Cricetidae, Sciuridae, and Heteromyidae. Specific representatives that we captured from these family groups fall into 3 distinct functional groups: nocturnal generalists (Cricetidae), diurnal generalists (Sciuridae), and nocturnal specialists (Heteromyidae). We quantified the variation in the abundance of each functional group explained by the percentage of cheatgrass cover using additional linear regression analyses (again using a negative binomial distribution for count data).

We also regressed species-specific abundances of small mammals as a function of the percentage of cheatgrass cover. These analyses excluded species that were found at less than 5% of sites. After analyzing the species-specific abundance data, we re-ran the overall abundance regression, but excluded small-mammal species that showed significant declines in the individual analyses. The purpose of this additional analysis was to determine if the declining but non-significant trends exhibited by many species was a significant decline when pooled. Similarly, we wanted to determine if the decline in overall small-mammal abundances was only significant because a few species were experiencing serious declines while most populations were stable. Because each of these analyses included count data, we continued to assume a negative binomial distribution for the error structure.

To assess the diversity of the small-mammal community at each site we used the exponential form of the Shannon-Wiener index (a measure more sensitive to species richness), and the reciprocal form of Simpson's index (a measure more sensitive to species evenness) [34]. Similarly, to allow us to account for the loss of plant diversity concurrent with cheatgrass invasion when modeling small-mammal abundances, we calculated these indices for the plant community. We used the Shannon and inverse Simpson functions within the vegan package in Program R to calculate these indices [35], [36]. We examined the indices relative to the percentage of cheatgrass cover at each site using linear regression in program R [33]. To meet normality assumptions of regression, we square-root transformed these indices for the small-mammal community. Additionally, regression models included sampling year to account for annual differences in capture rates.

Lastly, we used linear regression analyses (with a negative binomial distribution) to assess whether changes in the small-mammal community are due to a direct or indirect effect of cheatgrass invasion. We used regression equations that modeled abundances of small mammals as a function of capture year, the percentage of shrub cover, the diversity of the plant community, and the percentage of cheatgrass cover. We verified that these variables were suitable for inclusion in the same model by examining collinearity between variables. This allowed us to determine if cheatgrass cover impacted abundances after accounting for the decrease in shrub cover (resulting from increased frequency of fire associated with invasion of cheatgrass) and the decrease in the diversity of the plant community that occurred with increased cheatgrass. If cheatgrass cover did not significantly explain abundance after accounting for shrub cover and diversity of the plant community, our analyses would indicate that changes in abundance of small mammals were primarily indirect effects of invasion (e.g., fire, loss of overhead cover, or loss of plant diversity). However, if the percentage of cheatgrass cover remained a significant variable explaining variation in abundance of small mammals after accounting for these parameters, our analyses would indicate a direct negative impact of cheatgrass invasion on small mammals (e.g., inherent effects such as decreased food availability or decreased ability to move across the landscape through matted grass stems). We set alpha to be equal to 0.05 for all analyses.

## Results

We detected 113 plant species across the 70 sites we sampled. The percentage of cheatgrass cover ranged from 0–94% at these sites. As the percentage of cheatgrass cover increased, shrub cover (a variable strongly influenced by fire in Great Basin Desert shrub communities) significantly declined (estimate  = −0.201, SE = 0.051, t_67_ = −3.983, P<0.001). This change is indicative of the negative effects of increased fire frequency (caused by cheatgrass) on abundance of shrubs (i.e., at high densities of cheatgrass the shrub layer is often absent or diminished). Both the exponential Shannon-Wiener (estimate  = −4.683, SE = 0.8440, t_67_ = −5.549, P<0.001) and the reciprocal Simpson's (estimate  = −0.365, SE = 0.070, t_67_ = −5.187, P<0.001) indices (diversity of the plant community) also decreased with increased cover of cheatgrass.

We captured 580 unique small mammals during 10,437 trap nights. Individuals captured included representatives from 12 species, 10 genera, and 3 families (Table 1). In decreasing order of abundance, we captured: deer mouse (*Peromyscus maniculatus*), Ord's kangaroo rat (*Dipodomys ordii*), chisel-toothed kangaroo rat (*Dipodomys microps*), white-tailed antelope squirrel (*Ammospermophilus leucurus*), long-tailed pocket mouse (*Chaetodipus formosus*), Great Basin pocket mouse (*Perognathus parvus*), northern grasshopper mouse (*Onychomys leucogaster*), desert woodrat (*Neotoma lepida*), little pocket mouse (*Perognathus longimembris*), montane vole (*Microtus montanus*), least chipmunk (*Tamias minimus*), and dark kangaroo mouse (*Microdipodops megacephalus*; Table 1). We did not capture any small mammals at four sites (2 sites with relatively low percentage of cheatgrass cover and 2 with relatively high percentage of cheatgrass cover); therefore, these sites were excluded from subsequent analyses because indices of diversity could not be calculated. Because these sites were evenly split between low and high percentage of cheatgrass cover their removal was unlikely to have significant effects on our analysis. Of the remaining 66 sites, the number of individuals captured at each site ranged from 1 to 31.

**Table 1:** Abundance of Small Mammals.

	Cheatgrass Cover (%)			
Family *Species*	Low (0–1.66)	Medium-Low (1.67–10)	Medium-High (10–47)	High (47–100)
Heteromyidae				
*Dipodomys ordii*	3.00±1.19	1.47±0.75	2.38±0.66	2.71±1.25
*Dipodomys microps*	1.59±0.58	0.82±0.30	1.31±0.44	0.12±0.12
*Chaetodipus formosus*	0.35±0.35	0.82±0.49	1.13±0.62	0.18±0.13
*Perognathus parvus*	-	0.12±0.12	0.44±0.26	0.35±0.35
*Perognathus longimembris*	-	0.24±0.14	-	-
*Microdipodops megacephalus*	0.06±0.06	-	-	-
Cricetidae				
*Peromyscus maniculatus*	4.29±0.91	4.29±1.35	3.31±0.93	2.29±0.75
*Onychomys leucogaster*	0.06±0.06	0.18±0.10	0.19±0.14	-
*Neotoma lepida*	0.24±0.14	-	0.06±0.06	-
*Microtus montanus*	-	-	0.13±0.13	-
Sciuridae				
*Ammospermophilus leucurus*	1.24±0.57	0.76±0.41	0.38±0.22	0.06±0.06
*Neotamias minimus*	-	0.12±0.12	-	-
Total	10.82±1.80	8.82±1.41	9.31±1.19	5.71±1.40

Note. – Mean abundance of small mammals (minimum number known alive) captured by species ± SE in each quartile of cheatgrass cover (%) for 66 sites in the Great Basin Desert, Utah (data collected in 2011–2012).

**Figure 2:**
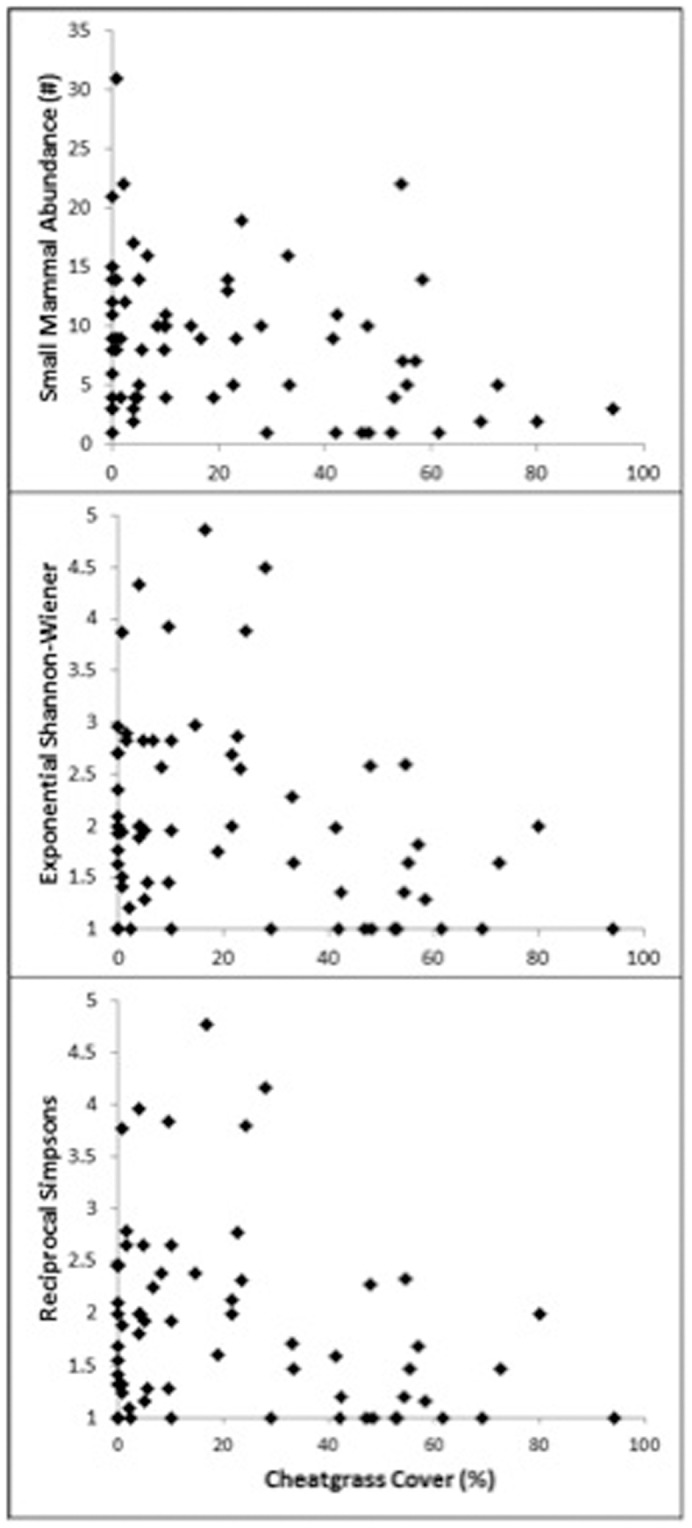
Cheatgrass and the Small-Mammal Community. Abundance (top), Exponential Shannon-Wiener index (middle), and Reciprocal Simpson's index (bottom) for small-mammal assemblages at 66 sites with varying cheatgrass (*Bromus tectorum*) cover in the Great Basin Desert, Utah, 2011–2012.

The overall abundances of small mammals had a negative slope when regressed against the percentage of cheatgrass cover (estimate  = −1.007, SE = 0.348, z_67_ = −2.893, P = 0.004; Figure 2). When the small-mammal assemblages were partitioned into functional groups, only one group, Sciuridae, had a significant decrease in abundance with increased cover of cheatgrass (estimate  = −4.968, SE = 1.915, z_67_ = −2.595, P = 0.009); neither Cricetid (estimate  = −0.870, SE = .537, z_67_ = −1.621, P = 0.105) nor Heteromyid (estimate  = −0.994, SE = 0.600, z_67_ = −1.656, P = 0.098) abundances decreased significantly (although there were negative trends for both groups). When the data were examined at the species level, 2 of 8 species decreased significantly in abundance with increased cheatgrass: Chisel-toothed kangaroo rat and white-tailed antelope squirrel (Table 2). After removing the chisel-toothed kangaroo rat and white-tailed antelope squirrel from the small-mammal community, there remained a negative trend in abundance with increased cheatgrass cover (estimate  = −0.658, SE = 0.365, z_67_ = −1.801, P = 0.072), suggesting that the decreasing trend in small mammals was not solely due to declines by these two species.

**Table 2:** Regression Coefficients.

Species	estimate	SE	z_67_ a	P^b^
*Chaetodipus formosus*	−1.242	0.817	−1.521	0.128
*Neotoma lepida*	−4.854	4.287	−1.132	0.258
*Peromyscus maniculatus*	−0.786	0.570	−1.379	0.168
*Onychomys leucogaster*	−2.209	2.218	−0.996	0.319
*Perognathus parvus*	1.821	1.644	1.107	0.268
*Dipodomys microps*	−3.077	1.118	−2.752	0.006*
*Dipodomys ordii*	−0.531	0.925	−0.574	0.566
*Ammospermophilus leucurus*	−4.759	1.942	−2.451	0.014*

Note. – Linear regression coefficients and standard errors (SE) for each species when small-mammal abundance is calculated as a function of the percentage of cheatgrass cover for sites in the Great Basin Desert, Utah (data collected 2011–2012). The asterisk denotes significance at the P<0.05 level.

a test statistic.

b p-value.

The diversity of the small-mammal community decreased with increased cover of cheatgrass. Both the square root-transformed exponential Shannon-Wiener (estimate  = −0.345, SE = 0.152, t_63_ = −2.263, P = 0.027) and the reciprocal Simpson's index (estimate  = −0.312, SE = 0.146, t_63_ = −2.139, P = 0.036) decreased with increased cover of cheatgrass (Figure 2). Additionally, the negative relationship between cheatgrass and small-mammal abundance remained significant even after accounting for declines in shrub cover (likely associated with fire) and plant diversity (estimate  = −1.464, SE = 0.482, z_64_ = −3.041, P = 0.002), indicating that there is a direct negative effect of cheatgrass invasion on small-mammal communities in the Great Basin.

## Discussion

Changes in the percentage of cheatgrass cover were associated with changes in the small-mammal community of the Great Basin Desert. These changes likely resulted from a decrease in habitat suitability when cheatgrass invaded [19]. Overall abundance, richness, and evenness of the small-mammal community decreased with increasing cover of cheatgrass. Additionally, there were significant declines in abundance (with increasing cheatgrass cover) of two small-mammal species and one functional group when analyzed individually. Similarly, the combined abundances of all species, excluding the two species (chisel-toothed kangaroo rat and white-tailed antelope squirrel) with a significant decline, was negatively associated with the percentage of cheatgrass cover. This finding indicates that decreases in overall community abundances were not entirely driven by the two species exhibiting statistically significant declines. In other words, there is likely a biologically significant decrease in the abundance of other species that our analysis did not detect due to sample size limitations. Additional samples of some species may have illustrated patterns more clearly, but the stratified random nature of our selection process prevented the purposeful selection of additional sites likely to be inhabited by certain species.

Changes in small-mammal abundance (overall and species-specific) and diversity often result from decreased niche availability [19], [37]. Niche selection enables otherwise similar small-mammal species to coexist and several partitioning theories may explain changes in abundance and diversity of small mammals [26]. These theories include the spatial partitioning of resources (e.g., Ord's kangaroo rats prefer to forage in open areas, while pocket mice generally forage under shrubbery or other cover) and the partitioning of food resources by seed size [26], [38]. Cheatgrass decreases both open space and shrub cover in a system, potentially negatively affecting these species [24]. Similarly, the decrease in shade availability and increase in restrictions on mobility that are concurrent with invasion by cheatgrass may impact small-mammal communities [39], [40]. The observed changes in the Great Basin likely indicate that species respond differently to cheatgrass invasion and that cheatgrass invasion has reduced the number of different niches available to partition.

While changes in microhabitat availability may explain some of the trends in small-mammal populations, changes in the food supply may explain others. For example, small mammals prefer native seed over that of cheatgrass, which is relatively low in calories and protein [25]. Foliage is also an important food source for small mammals and is altered by cheatgrass invasion. For example, deer mice are known to increase consumption of foliage when there is a lack of precipitation, presumably because it is a source of moisture [41]. Additionally, there is a correlation between ingestion of green vegetation and reproductive activity in small mammals [42]. Because cheatgrass is green for only a short time when moisture is available [10], the lack of available green vegetation throughout the summer where cheatgrass is a dominant plant may impact survival or reproduction in small-mammal populations, contributing to the reduced abundance and diversity that we witnessed.

These potential mechanisms of small mammal decline may be linked directly or indirectly to cheatgrass invasion. Direct effects likely include a reduction in quality forage and increased obstruction to mobility [25], [40]. Examples of indirect effects of cheatgrass include changes in the native herbaceous plant community, fire, and the associated reduction in shrub species [24]. The percentage of cheatgrass cover remained a significant correlate of small-mammal abundance even after accounting for changing shrub cover and decreasing diversity of the plant community. This regression accounts for likely indirect effects, indicating that cheatgrass was likely directly affecting small-mammal abundance (in addition to indirect effects). Although the fire history at our sites was unknown, shrub cover was a plausible substitute [24].

The described changes in the small-mammal community may have effects at both higher and lower trophic levels. For example, several desert canid species are dependent on small mammals for the majority of their diet and changes in the small-mammal community may impact these populations (e.g., kit fox populations have declined following declines in small-mammal abundance) [43]. Additionally, many avian predators (raptors) in the Great Basin depend on small mammals for the majority of their diet [44], [45] and changes in small-mammal abundance have been associated with changes in population size of some raptor species [46]. As a primary prey source, the significant decline in small-mammal abundance that occurred with increasing cover of cheatgrass may have negative effects on both diurnal and nocturnal species of raptors.

Removal or modification of top-down pressures can also have extreme effects on desert plant communities. Experimental removal of kangaroo rats from plots in the Chihuahuan Desert revealed that grasses released from pressures exerted by kangaroo rats had increased leaf and tiller growth and inflorescence production [47]. Other plots exhibited a three-fold increase in annual and perennial grass density where kangaroo rats were experimentally removed [28]. This change was dominated by a 20-fold increase in a single perennial grass species. In addition to direct effects of cheatgrass invasion on plant communities, the decline in abundance of small mammals – and one species of kangaroo rat in particular –associated with invasion likely has additional top-down influences on native vegetation.

Our analysis indicates that cheatgrass invasion is associated with changes in the small-mammal community. Ostoja and Schupp [19] reported similar findings but wondered whether their localized results were applicable to the Great Basin as a whole (they believed that they were). Our results build on that previously available as we collected data from sites across a broad spatial scale and spectrum of cheatgrass invasion. Additionally, we made several measurements of the plant community at each site, allowing our analyses to account for variation in the plant community and the presence of fire. Our results indicated that cheatgrass decreased the diversity and abundance of small mammals at invaded sites across a large portion of the Great Basin. Moreover, changes in the small-mammal community are likely the result of both direct (e.g., decreases in niche/food availability or increased mobility restrictions) and indirect effects (e.g., decreased shrub cover and increased fire frequency) associated with cheatgrass invasion. Negative effects of invasion on the small-mammal community will reduce food availability for higher trophic levels and remove a top-down pressure in this system, likely modifying plant population dynamics and resulting in a system regulated by bottom-up forces (cheatgrass). As the invasion of cheatgrass continues to spread across the western United States, implications for both plant and animal biodiversity and ecosystem function are severe.
